# Outpatient healthcare costs associated with overweight and obesity in Italy

**DOI:** 10.1186/s12913-023-09576-4

**Published:** 2023-06-12

**Authors:** Vincenzo Atella, Federico Belotti, Claudio Cricelli, Matilde Giaccherini, Gerardo Medea, Antonio Nicolucci, Andrea Piano Mortari, Paolo Sbraccia

**Affiliations:** 1 Department of Economics and Finance, University of Rome Tor Vergata, via Columbia n. 2, 00133, Roma, Italy; 2 CEIS Tor Vergata, University of Rome Tor Vergata, Roma, Italy; 3 Italian College of General Practitioners, Florence, Italy; 4 Department of Systems Medicine, University of Rome Tor Vergata, Roma, Italy; 5 Center for Outcomes Research and Clinical Epidemiology – CORESEARCH, Pescara, Italy; 6 Department of Programming, Ministry of Health, Roma, Italy

**Keywords:** Obesity, Healthcare costs, Comorbidities

## Abstract

**Background:**

To evaluate outpatient healthcare expenditure associated with different levels of BMI and glucose metabolism alterations.

**Methods:**

The study is based on a representative national sample of adults, with data obtained from electronic clinical records of 900 Italian general practitioners. Data relative to the year 2018 were analyzed. The study population was classified according to BMI (normal weight, overweight, and obesity classes 1, 2, and 3) and glucose metabolism status (normoglycemia – NGT; impaired fasting glucose – IFG; diabetes mellitus – DM). Outpatient health expenditures include diagnostic tests, specialist visits, and drugs.

**Results:**

Data relative to 991,917 adults were analyzed. Annual per capita expenditure rose from 252.2 Euro among individuals with normal weight to 752.9 Euro among those with class 3 obesity. The presence of obesity determined an excess cost, particularly among younger individuals. Within each BMI class, the presence of IFG or DM2 identified subgroups of individuals with substantially higher healthcare expenditures.

**Conclusions:**

Outpatient healthcare costs markedly increased with increasing BMI in all age categories, particularly among individuals below 65. Addressing the double burden of excess weight and hyperglycemia represents a significant challenge and a healthcare priority.

**Supplementary Information:**

The online version contains supplementary material available at 10.1186/s12913-023-09576-4.

## Bullet points

### What is already known about this subject?

- Excess weight is responsible for a high clinical, social, and economic burden

### What are the new findings in your manuscript?

- Costs associated with drugs and outpatient services markedly increase with BMI in all age categories and are significantly influenced by the concomitance of glucose metabolism alterations;
- The impact of overweight and obesity is particularly strong among younger individuals.

### How might your results change the direction of research or the focus of clinical practice?

- Our data emphasize the crucial role of primary prevention of obesity and hyperglycemia.
- These findings are particularly significant for primary care, representing the forefront of the fight against obesity, diabetes, and related comorbidities.

## Background

The rising prevalence of overweight and obesity is a worldwide cause of serious concern, and the phenomenon is increasingly becoming a global pandemic, having nearly tripled since 1975. Since 1980, the prevalence of obesity, defined as a body mass index (BMI) greater or equal to 30 kg/m^2^, has doubled in more than 70 countries and has risen continuously in many others [1]. According to the WHO [2], in 2016, more than 1.9 billion adults, i.e., aged 18 years and older, were overweight. Of these, over 650 million were obese. Furthermore, 39 million children under the age of 5 were either overweight or obese in 2020 and over 340 million children and adolescents aged 5–19 were overweight or obese in 2016. Most of the world’s population lives in countries where overweight (25$\leqslant$BMI < 30 kg/m^2^) and obesity are associated with higher mortality with respect to being underweight. The World Obesity Federation [3] projected that by 2030 about 1 in 5 women and 1 in 7 men would be living with obesity, summing up to over 1 billion people globally (it was 511 million in 2010).

Globally, more than 160 million lost years of healthy life were due to high BMI in 2019, and this figure is projected to increase each passing year [4]. This is more than 20% of all lost years of healthy life caused by preventable chronic ill-health. Since obesity is preventable and a major risk factor for many chronic conditions, it is necessary to manage high BMI to reduce preventable non-communicable diseases [5]. High body mass index caused 4.0 million deaths globally, nearly 40% of which occurred in non-obese people (BMI < 30 kg/m^2^). More than two-thirds of high BMI-related deaths were due to cardiovascular disease [1].

Excess weight is responsible for a high clinical, social, and economic burden linked to the numerous non-communicable diseases associated with overweight and obesity, including diabetes, cardiovascular diseases, respiratory diseases, some types of cancer, osteoarticular diseases, and depression [6]. Excess weight is also correlated with increased health care services utilization, leading to higher direct medical costs, especially for patients with severe obesity [7].

The higher utilization of health care services by patients with excess body weight is mainly driven by comorbidities: they consume more primary care and outpatient specialty care visits, more hospitalizations, undergo more surgeries and use more diagnostic and home health care services [8–10]. People with obesity also receive 2.4 times more prescriptions than people with a normal weight [8].

According to OECD estimates, on average, 8.4% of total health care spending is devoted to treating the diseases caused by obesity [11]. In EU28 countries, the average annual per capita cost attributable to overweight amounts to 195.4 PPP USD, corresponding to 8.0% of total healthcare expenditures, while in Italy, per capita cost amounts to 234 PPP USD, corresponding to 9.0% of the total. Excess body weight is responsible, on average, for 70% of all treatment costs for diabetes, 23% of treatment costs for cardiovascular diseases, and 9% for cancers [11]. The latest data from World Obesity Federation [3] estimated that the global medical cost to treat obesity-related diseases will reach $1.2 trillion annually by 2025. Therefore, promoting initiatives to tackle obesity remains an important policy goal.

In a large, population-based study in primary care in Italy, we have previously shown that the interplay between excess weight and glucose metabolism alterations increases the likelihood of other chronic conditions [12]. The present study aimed to evaluate outpatient healthcare expenditure associated with different degrees of overweight in the same population and the impact of the interplay between obesity and glucose metabolism alterations on healthcare expenditures.

## Methods

The study is based on data from the Health Search/IQVIA Health LPD Longitudinal Patient Database (HS), an Italian general practice registry, containing data from electronic clinical records (ECRs) of patients aged 14 + registered with a group of 900 General Practitioners (GPs), distributed across all Italian regions to be representative of the Italian population of GP’s patients. GPs voluntarily agree to collect clinical data and attend training courses for data entry [13]. Furthermore, in order to be considered for participating in epidemiological studies, all recruited GPs need to meet “up to standard” quality criteria relative to the levels of coding, the prevalence of well-known diseases, mortality rates, and years of recording activity [13]. The database complies with European Union guidelines on using medical data for research and has been previously demonstrated to be a valid data source for scientific research [14]. To guarantee the quality of the data and the reliability of the results, only patients who were still alive and had not revoked their GP were considered. A key feature of the HS database is that it includes all patients registered in the GP rosters, thus avoiding selection bias based on health status. Each person resident in Italy, irrespective of citizenship, is enrolled with a GP who acts as a gatekeeper for the system, and there is no private GP sector. Thus, GP’s electronic clinical records guarantee the presence of information about all individuals registered with a GP, regardless of their health status. Of note, the Italian National Institute of Statistics (ISTAT) uses these data to complement the information collected with the annual national health survey [15]; moreover, the Italian Drug Agency has routinely used the Health Search database as a source for the National Report on the Use of Drugs in Italy since 2004 [16, 17]. Furthermore, researchers have observed “a high degree of overlap between the population represented in the Health Search database with respect to what is reported by ISTAT” [18].

The database contains patient demographic data (age, sex, province of residence) that are linked through an encrypted patient code with their medical records (diagnoses, prescribed tests, and tests results), drug prescription information (medication name, date of filled prescription, and the number of days of supply), self-reported hospital admissions, and date of death. Although GPs collect the information daily, for this analysis, the information has been aggregated at the year level. Whenever information on specific variables was recorded more than once in a year (i.e., diagnostic test values and BMI levels), we have averaged these values over the year, thus resulting in a single observation per year.

In 2018, the last available sample in our possession, the original sample contained 1,006,920 individuals. For our study, we selected people aged 18–95 years, thus leading to a sample of 991,920 individuals. Finally, we have discarded outliers and mis-recordings of diagnostic test variables (i.e., negative and implausible values). This selection process produced a final sample of 991,917 individuals.

Since the dataset presented missing data in some variables of interest for our study, statistical imputation was applied. For this reason, although the analyses for this report were conducted only on information referring to the year 2018, we exploited the longitudinal features of the sample that allowed us to impute some of the missing data appropriately. The details of the imputation techniques adopted are reported in appendix 1.

The study population was classified according to BMI levels (expressed as kg/m^2^) in the following groups: normal weight (BMI between 18.5 and 24.99), overweight (BMI between 25 and 29.99), obesity class 1 (BMI between 30 and 34.99), obesity class 2 (BMI between 35 and 39.99), obesity class 3 (BMI ≥ 40).

The population was also classified according to glucose metabolism status into three classes: normal glucose tolerance (NGT; no diagnosis of diabetes mellitus over the period 2004–2018, fasting blood glucose < 100 mg/dl), impaired fasting glucose (IFG; no diagnosis of diabetes mellitus over the period 2004–2018, fasting blood glucose between 100 and 125 mg/dl), type 2 diabetes mellitus (DM2; diagnosis of type 2 diabetes mellitus over the period 2004–2018).

All diagnoses are coded according to the International Classification of Disease, Ninth Revision (ICD-9 CM) [19]. The list of codes used to identify comorbidities is reported in appendix 2. Among neoplasms, those affecting the digestive apparatus were selected (esophagus, stomach, intestine, colon, rectum, liver, gallbladder, pancreas).

As we deal with data collected by general practitioners, health expenditure data refers to total outpatient expenditure, which includes spending on diagnostic tests, specialist visits, and drugs. Expenditure data are extremely precise as we have been able to assign prices and tariffs to each single drug package (price), diagnostic tests (tariff) and specialist visit (tariff) prescribed by each GP to each patient. Therefore, the total expenditure by patient is simply obtained as the sum of all prescribed item expenditures. Expenditure is expressed in euro in annual terms. It is worth highlighting that although we found a strong cost gradient between normal-weight patients and patients with different levels of excess weight, this gradient represents an underestimation of the actual value due to lack of inpatient expenditures (mostly costs due to hospital admissions).

Data are summarized as mean and standard deviation (continuous variables) or percentages (categorical variables).

## Results

### Population characteristics

Overall, data relative to 991,917 adults were analyzed. The prevalence of overweight was 39.4%, while the prevalence of obesity was 11.1% (class 1: 7.9%, class 2: 2.3%, class 3: 0.9%). Participants’ characteristics, overall and by BMI classes, are reported in Table 1. Among individuals with excess weight, those with class 3 obesity had the lowest average age, while those with obesity class 1 tended to be older. The prevalence of female gender increased with increasing BMI, from 38.8% among overweight individuals to 66.8% among individuals with severe obesity.

Average values of fasting blood glucose, HbA1c, blood pressure, and triglycerides monotonically increased with BMI. The prevalence of glucose metabolism alterations markedly increased with BMI. Compared to individuals with normal weight, the prevalence of DM2 was five times higher among those overweight, ten times higher among those with obesity class 1, 13 times higher among those with obesity class 2 and 15 times higher among individuals with obesity class 3. Overall, the prevalence of IFG was 4.2%, being the lowest in individuals with normal weight (2.4%) and the highest among those with obesity class 1 (7.9%). The prevalence of major cardiovascular risk factors and cardiovascular events was also associated with BMI. In particular, the proportion of people affected by hypertension monotonically increased with BMI to 72.5% among individuals with severe obesity. Compared to people with normal weight, the prevalence of coronary heart disease was four times higher among overweight people and 5.5 times higher among obesity class 1 individuals. At the same time, it slightly decreased among individuals with very severe obesity. Similarly, the prevalence of cerebrovascular disease was about three times higher in individuals with any excess weight level, the highest among those with obesity class 1. The prevalence of heart failure dramatically increased with BMI levels; in particular, compared to normal weight, obesity class 3 was associated with ten times higher prevalence of heart failure.

**Table 1:** Population characteristics, overall and by BMI classes

Characteristics	Overall	Normal weight	Overweight	Obesity class 1	Obesity class 2	Obesity class 3
N	991,917	476,571	390,979	78,413	22,887	8,752
Age (years)	52.5 ± 18.7	44.5 ± 17.3	60.30 ± 16.5	62.0 ± 15.8	60.6 ± 15.7	58.2 ± 15.4
Gender (% females)	51.9	61.8	38.8	48.1	59.0	66.8
Fasting blood glucose (mg/dl)	102.6 ± 28.3	94.3 ± 20.8	104.8 ± 28.1	113.0 ± 34.3	116.9 ± 38.3	118.7 ± 40.3
HbA1c (mmol/mol)	45.2 ± 12.8	42.5 ± 12.1	45.2 ± 12.5	46.8 ± 13.9	47.9 ± 13.9	47.4 ± 14.4
Systolic blood pressure (mmHg)	131.3 ± 14.3	127.8 ± 14.6	132.6 ± 13.8	134.0 ± 13.5	134.7 ± 13.6	134.9 ± 14.1
Diastolic blood pressure (mmHg)	78.4 ± 8.3	77.1 ± 8.3	78.8 ± 8.1	79.5 ± 8.1	80.1 ± 8.2	80.8 ± 8.5
Total cholesterol (mmol/l)	5.13 ± 1.01	5.23 ± 0.99	5.13 ± 1.01	4.99 ± 1.03	4.92 ± 1.02	4.86 ± 0.95
HDL cholesterol (mmol/l)	1.45 ± 0.38	1.58 ± 0.40	1.40 ± 0.35	1.32 ± 0.33	1.30 ± 0.32	1.29 ± 0.32
LDL cholesterol (mmol/l)	3.08 ± 0.88	3.14 ± 0.87	3.08 ± 0.89	2.97 ± 0.89	2.90 ± 0.88	2.87 ± 0.90
Triglycerides (mmol/l)	1.38 ± 0.85	1.20 ± 0.76	1.44 ± 0.87	1.63 ± 0.96	1.63 ± 0.96	1.60 ± 0.87
Glycemic status (%)						
NGT	86.9	95.2	82.4	67.6	61.7	57.7
IFG	4.2	2.4	5.6	7.9	6.8	5.4
DM	8.9	2.4	12.0	24.5	31.5	36.8
Hypertension (%)	32.9	15.5	45.4	65.2	70.0	72.5
Dyslipidemia (%)	20.3	11.9	27.2	34.7	31.5	26.4
Coronary heart disease (%)	1.95	0.74	2.95	4.09	3.72	2.45
Cerebrovascular disease (%)	5.16	2.63	7.15	9.82	8.78	7.16
Heart failure (%)	4.10	1.43	5.50	10.32	12.44	14.05
Osteoarticular diseases* (%)	6.87	2.81	9.41	15.16	18.05	19.10
Depression (%)	6.18	4.78	7.05	8.67	9.57	11.09
Chronic kidney disease (%)	2.37	1.11	3.00	5.65	6.00	6.11
Cancer**(%)	1.12	0.81	1.36	1.72	1.44	1.03
Sleep apnea (%)	1.67	0.59	1.88	4.52	7.62	11.95
PCOS*** (%)	1.49	1.87	0.77	1.15	1.30	1.59
No. of comorbidities (%)						
0	59.2	77.4	45.8	27.1	23.1	20.8
1–3	39.2	22.0	52.2	68.5	71.5	73.0
> 3	1.6	0.6	2.0	4.4	5.5	6.2

* Hip and knee osteoarthrosis

** Esophagus, stomach, intestine, colon, rectum, liver, gallbladder, pancreas

*** Polycystic ovary syndrome

Among the other chronic conditions considered, osteoarticular diseases, depression, chronic kidney disease, and sleep apnea markedly increased with BMI.

### Outpatient healthcare expenditure

The average annual per capita outpatient healthcare expenditure was 398.6 Euro (range 0–3726 Euro). The average payment for drugs was 241.0 Euro (range 0–2928 Euro), while costs related to diagnostic tests and specialist visits amounted to 143.6 Euro (range 0–1503 Euro).

**Table 2:** Population characteristics according to quartiles of outpatient healthcare expenditure

Characteristics	1st quartile	2nd quartile	3rd quartile	4th quartile
**Average expenditure (€)**	3.80	101.51	320.31	1184.11
	(13.35)	(92.29)	(213.17)	(748.30)
**Age (years)**	44.0	48.7	54.1	64.0
	(16.1)	(17.2)	(18.3)	(16.9)
**Gender (% females)**	41.2	47.5	59.2	60.6
**BMI**				
Normal weight	56.0	40.0	49.0	49.0
Overweight	34.0	47.0	39.0	39.0
Obesity class 1	7.0	9.0	8.0	8.0
Obesity class 2	2.0	3.0	2.0	2.0
Obesity class 3	1.0	1.0	1.0	1.0
**Glycemic status (%)**				
NGT	95.5	91.7	85.1	71.2
IFG	0.4	3.1	5.6	8.2
DM	4.0	4.7	8.2	18.8
**Hypertension (%)**	13.2	25.0	38.3	57.0
**Dyslipidemia (%)**	8.5	15.2	22.9	35.6
**Coronary heart disease (%)**	0.77	0.47	1.38	5.16
**Cerebrovascular disease (%)**	1.57	1.89	4.60	12.63
**Heart failure (%)**	1.18	1.29	3.69	10.26
Osteoarticular diseases* (%)	2.22	4.42	7.69	13.50
**Depression (%)**	3.09	4.08	6.47	11.23
**Chronic kidney disease (%)**	1.11	0.71	1.89	5.71
Cancer**(%)	0.56	0.40	0.92	2.56
**Sleep apnea (%)**	0.85	1.30	1.77	2.82
PCOS*** (%)	1.67	1.66	1.63	1.10
**No. of comorbidities (%)**				
0	82.02	68.25	53.08	31.42
1	13.21	24.69	31.15	33.66
2	3.01	5.70	11.58	21.10
≥ 3	1.11	1.11	3.24	9.36

* Hip and knee osteoarthrosis

** Esophagus, stomach, intestine, colon, rectum, liver, gallbladder, pancreas

*** Polycystic ovary syndrome

Population characteristics according to quartiles of expenditure are reported in Table 2. The mean age and percentage of women increased with increasing cost. The prevalence of glucose metabolism alterations markedly increased across quartiles of expenditure: in the upper quartile, about 30% of the study population had either IFG or DM2. Similarly, the prevalence of hypertension, dyslipidemia, and all comorbidities considered, except for PCOS, increased with expenditure. In the upper quartile of expenditure, about 70% of the study population had one or more of the comorbidities considered, and one in ten had three or more comorbidities.

**Table 3:** Average outpatient healthcare expenditure by BMI classes

Expenditure	Normal weight	Overweight	Obesity class 1	Obesity class 2	Obesity class 3
**Total**	**252.22**	**494.85**	**692.12**	**734.39**	**752.90**
**Drugs** (overall)	**130.25**	**316.59**	**454.00**	**492.35**	**504.49**
ATC Classes*					
Class A	18.01	45.32	67.73	75.77	78.60
Class B	8.20	24.94	35.69	37.35	40.57
Class C	25.54	86.78	133.74	143.47	145.59
Class D	0.49	0.63	0.87	0.88	1.00
Class G	3.96	8.13	9.14	6.41	4.65
Class H	1.34	2.08	2.80	3.23	3.68
Class J	6.15	8.87	10.29	11.04	12.72
Class L	0.65	0.96	1.13	1.20	1.31
Class M	3.48	7.13	9.83	11.31	11.88
Class N	15.99	27.82	36.98	40.47	44.44
Class P	0.00	0.00	0.00	0.00	0.00
Class R	7.45	12.49	17.24	19.42	23.06
Class S	0.98	2.15	2.54	2.39	2.32
**Diagnostics – Visits**	**112.75**	**161.04**	**215.76**	**218.06**	**221.64**
Laboratory Diagnostics	33.25	44.44	59.60	61.77	64.08
Non-Laboratory Diagnostics	66.91	99.94	135.57	135.26	131.61
Check-up Visits	1.69	3.11	4.05	4.22	4.58
Specialist Visits	5.79	8.00	9.69	10.10	10.61

**Fig. 1:**
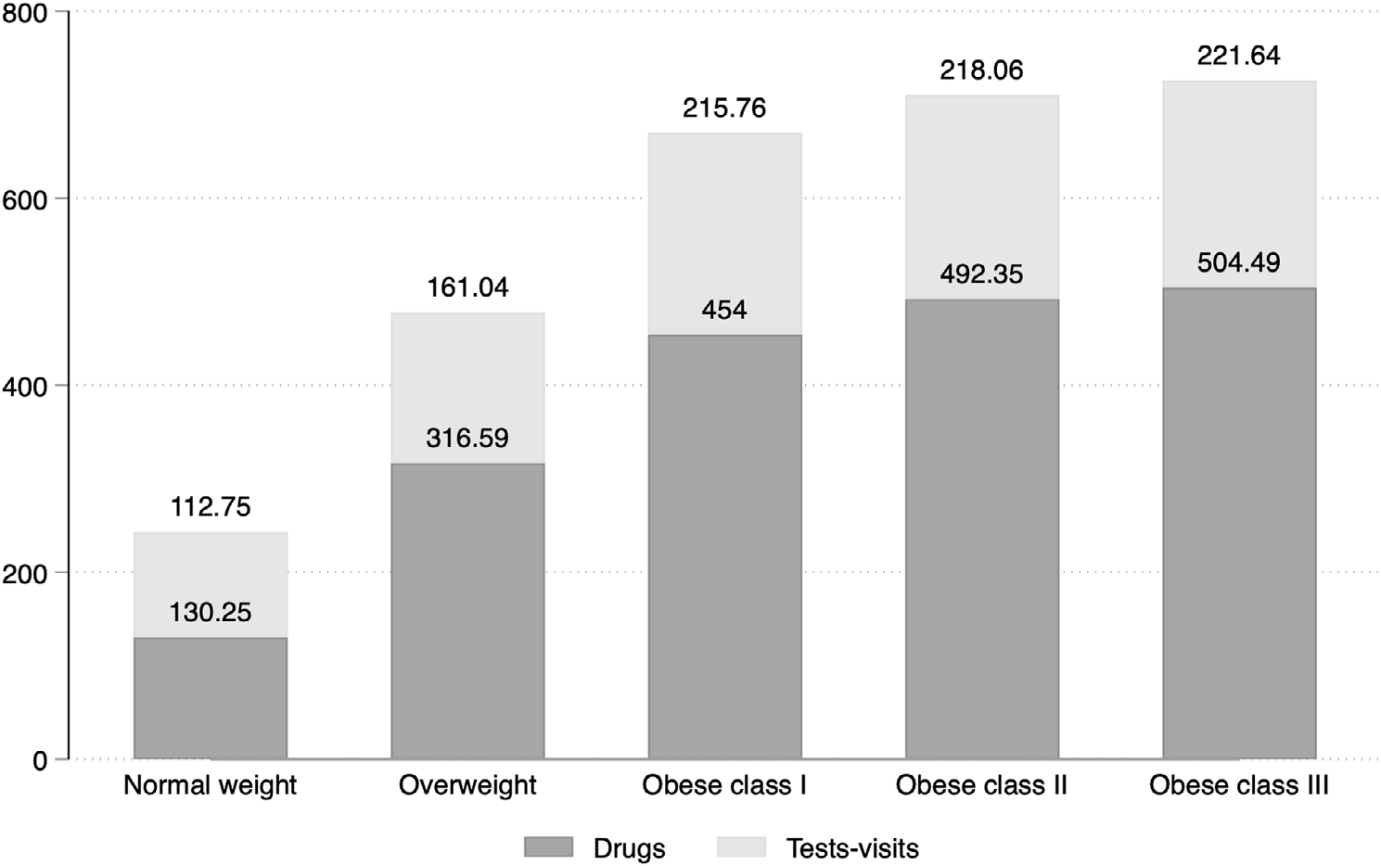
Average healthcare outpatient expenditure by BMI classes

Table 3 shows the annual per capita outpatient healthcare expenditure by BMI classes. Total costs rose from 252.2 Euro among individuals with normal weight to 752.9 Euro among those with class 3 obesity. Drug-related expenses and costs associated with visits and diagnostic tests linearly increased with BMI (Fig. 1). Among drug classes, the most significant contribution to costs was provided by drugs of the cardiovascular system (ATC class C), drugs of the alimentary tract and metabolism (ATC class A), and drugs of the nervous system (ATC class N).

**Fig. 2:**
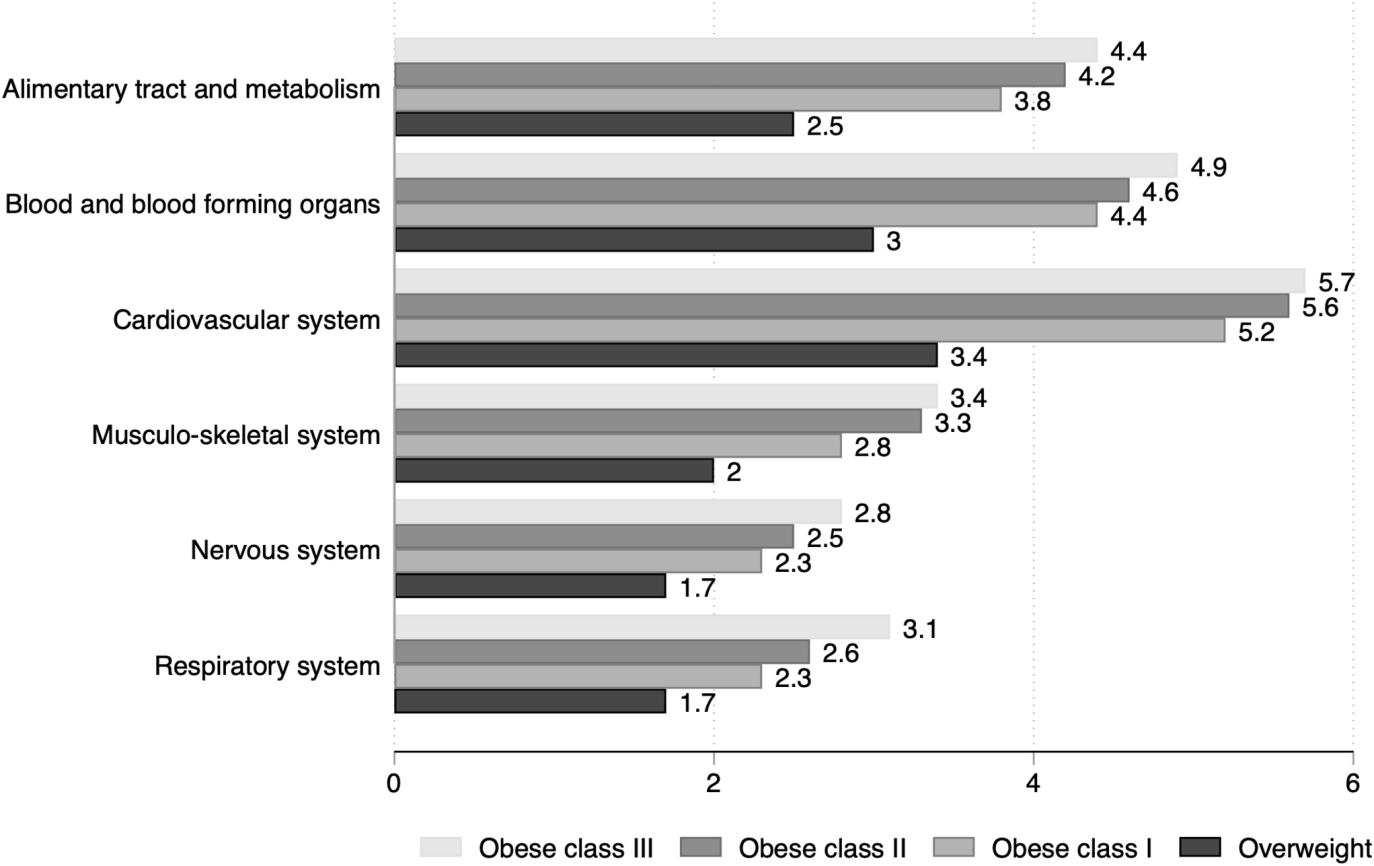
Expenditure ratio for selected ATC classes in individuals with overweight/obesity vs. normal weight

Compared to individuals with normal weight, the drug expenditure ratio increased with BMI; overweight was associated with an excess cost ranging from 70% (drugs of the nervous system and respiratory system) to more than threefold (medicines of the cardiovascular system) (Fig. 2). Expenditure ratio raised across BMI classes, and the cost for obesity class 3 was from 2.8 times (drugs of the nervous system) to 5.7 times higher (medicines of the cardiovascular system) compared to individuals with normal weight.

**Table 4:** Outpatient healthcare expenditure by BMI and age classes

Expenditure	Normal weight			Overweight			Obesity class 1			Obesity class 2			Obesity class 3		
	≤ 40	41–65	> 65	≤ 40	41–65	> 65	≤ 40	41–65	> 65	≤ 40	41–65	> 65	≤ 40	41–65	> 65
**Total expenditure**	**108.9**	**254.5**	**732.3**	**125.2**	**329.8**	**814.6**	**181.3**	**495.2**	**1,000**	**212.2**	**575.9**	**1,060**	**257.2**	**650.4**	**1,090**
**Drugs**	**41.79**	**118.9**	**466.2**	**57.87**	**187.5**	**556.9**	**78.18**	**297.0**	**692.8**	**104.5**	**360.8**	**750.2**	**117.5**	**422.7**	**770.0**
ATC Class A	4.34	16.39	69.48	7.37	26.25	80.74	8.37	42.15	106.2	12.98	52.77	119.5	17.35	62.52	125.2
ATC Class B	1.80	5.07	39.66	1.98	9.92	50.60	2.90	16.09	62.34	3.77	19.25	67.37	9.01	25.17	74.97
ATC Class C	1.12	21.42	121.4	3.78	53.31	154.2	8.29	94.32	200.6	13.08	111.3	216.3	15.74	122.8	227.9
ATC Class D	0.40	0.46	0.90	0.40	0.49	0.88	0.61	0.71	1.08	0.62	0.75	1.10	0.48	0.91	1.32
ATC Class G	2.32	3.76	10.17	1.21	4.63	14.63	2.37	5.14	14.59	3.05	3.93	10.18	1.92	3.47	7.40
ATC Class H	0.65	1.60	2.83	0.76	1.72	2.94	1.22	2.43	3.52	1.33	2.96	4.06	1.91	3.36	4.82
ATC Class J	4.99	6.41	9.32	5.72	7.84	11.15	6.92	9.07	12.25	7.40	10.17	13.04	8.74	12.10	15.11
ATC Class L	0.20	0.80	1.70	0.21	0.71	1.49	0.22	0.81	1.64	0.39	0.97	1.68	0.06	1.25	1.87
ATC Class M	1.01	3.87	10.64	1.45	5.09	11.46	2.30	6.93	14.38	2.70	8.78	16.60	2.97	9.78	18.32
ATC Class N	5.92	17.88	44.36	8.39	17.75	46.33	11.85	25.35	54.06	11.45	31.65	58.59	13.65	37.92	65.59
0.00ATC Class P	0.00	0.00	0.00	0.00	0.00	0.00	0.00	0.00	0.00	0.00	0.00	0.00	0.00	0.00	0.00
ATC Class R	5.05	7.52	15.38	6.18	9.40	18.29	8.87	12.38	23.90	10.03	14.75	27.38	9.03	19.31	33.83
ATC Class S	0.29	0.82	3.85	0.33	1.11	4.00	0.47	1.37	4.16	0.41	1.38	4.09	0.71	1.53	4.07
**Diagnostics - Visits**	**64.09**	**126.0**	**237.1**	**63.48**	**130.7**	**229.3**	**95.65**	**179.0**	**278.9**	**102.0**	**193.3**	**278.5**	**131.1**	**208.3**	**275.3**
Laboratory Diagnostics	22.21	35.19	64.75	19.33	34.87	64.15	30.38	48.14	77.44	36.31	52.17	79.81	44.22	59.33	78.52
Non-Laboratory Diagnostics	32.12	78.83	148.1	35.85	83.87	140.1	51.00	114.8	174.9	53.92	123.6	171.1	66.09	126.4	163.9
Check-up Visits	0.78	1.74	4.61	0.86	2.19	4.95	1.25	2.98	5.73	1.41	3.54	5.79	2.06	2.11	5.98
Specialist Visits	3.87	6.10	11.35	3.71	6.30	11.44	5.38	8.03	12.30	5.70	9.02	12.55	7.32	6.47	12.24

* Class A: alimentary tract and metabolism; class B: blood and blood forming organs; class C: cardiovascular system; class D: dermatologicals; class G: genito urinary system and sex hormones; class H: systemic hormonal preparations, excluding sex hormones and insulins; class J: antiinfective for systemic use; class L: antineoplastic and immunomodulating agents; class M: musculo-skeletal system; class N: nervous system; class P: antiparasitic products, insecticides and repellents; class R: respiratory system; class S: sensory organs.

Table 4 shows the annual per capita outpatient healthcare expenditure by age class and BMI category. Within each age class, expenses increased with BMI (figure S1); on the other hand, within each BMI class, costs markedly increased with increasing age.

**Fig. 3:**
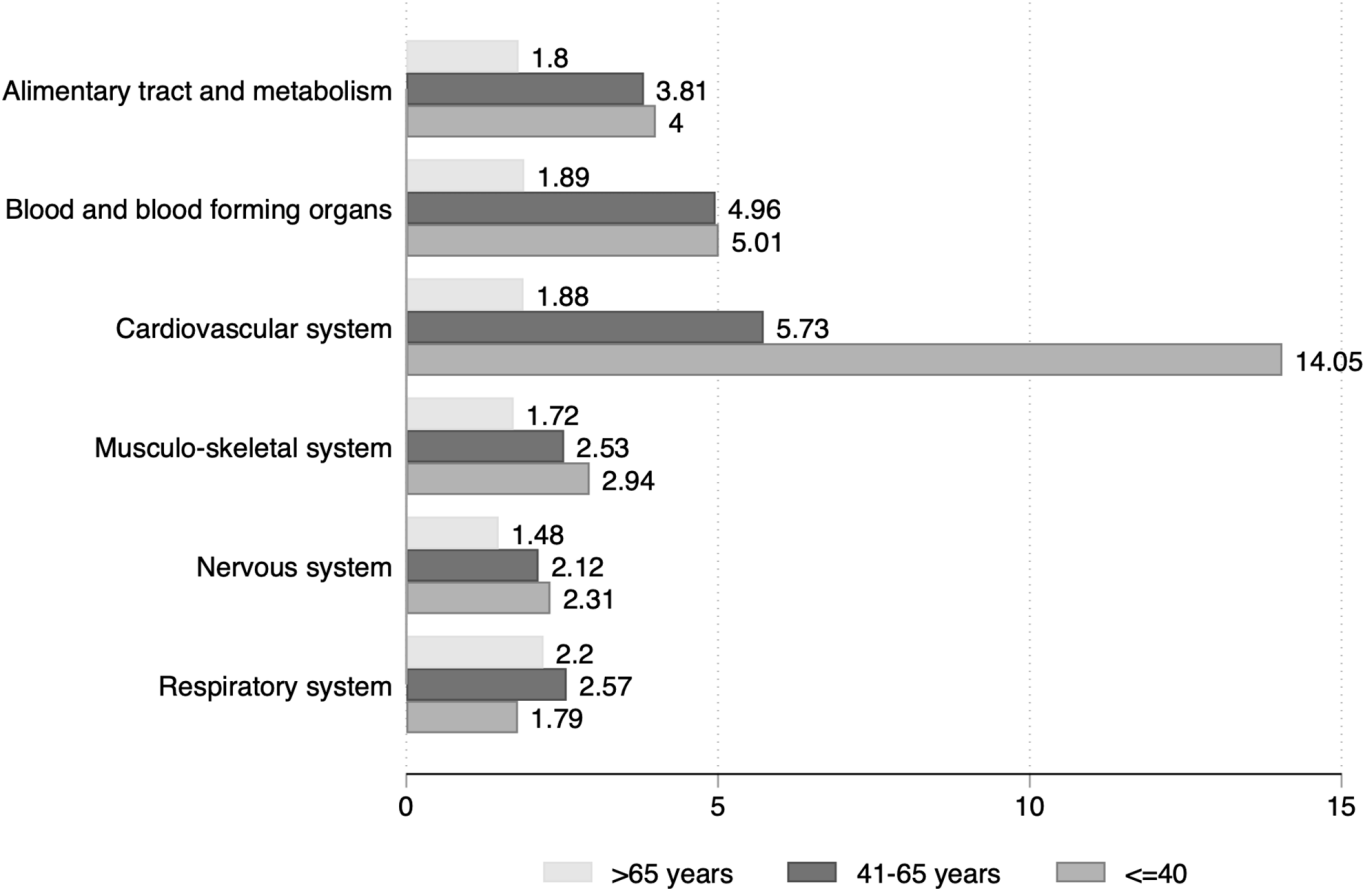
Expenditure ratio for selected ATC classes in individuals with obesity class 3 vs. normal weight, by age classes

A further interesting way of looking at these results is through the lenses of Fig. 3, where we focus on drug costs. The presence of obesity determined an excess cost particularly among younger individuals. In the age group ≤ 40 years, compared to normal-weight people, those with class 3 obesity had a fourteen times higher expenditure for drugs of the cardiovascular system (ATC class C), a fivefold higher payment for drugs relative to blood and blood-forming organs (ATC class B) and a fourfold increased cost for medications of the alimentary tract and metabolism (ATC class A) (Fig. 3). Drugs expenditure ratio between obesity class 3 and normal weight tended to decrease with increasing age, being in any case higher in individuals with obesity.

**Table 5:** Outpatient healthcare expenditure by BMI classes and glucose metabolism status

Expenditure	Normal weight			Overweight			Obesity class 1			Obesity class 2			Obesity class 3		
	NGT	IFG	DM	NGT	IFG	DM	NGT	IFG	DM	NGT	IFG	DM	NGT	IFG	DM
**Total expenditure**	**219.0**	**686.7**	**1,010**	**398.5**	**812.4**	**967.1**	**515.4**	**874.3**	**1,089**	**518.8**	**907.7**	**1,098**	**534.2**	**885.2**	**1,059**
**Drugs**	**108.2**	**352.4**	**710.7**	**247.7**	**477.6**	**690.1**	**323.4**	**523.9**	**772.0**	**328.2**	**533.2**	**791.9**	**343.5**	**508.7**	**744.6**
ATC Class A	13.06	42.49	175.8	28.03	52.48	157.2	33.64	54.96	162.9	33.94	51.76	160.3	35.19	56.17	148.3
ATC Class B	6.47	27.64	51.71	19.51	42.30	52.06	25.98	44.84	57.75	26	45.24	56.51	30.43	36.03	57.08
ATC Class C	19.85	94.40	163.2	68.14	148.2	179.1	98.36	172.7	212.2	100.7	179.2	214.3	100.1	166.0	210.3
ATC Class D	0.46	1.06	0.88	0.57	1.02	0.81	0.75	1.24	1.02	0.79	1.12	0.97	0.78	1.27	1.27
ATC Class G	3.56	9.70	12.79	6.82	14.99	13.29	7.26	12.98	12.63	5.05	7.10	8.78	3.66	5.55	5.91
ATC Class H	1.25	2.91	2.65	1.94	2.91	2.57	2.69	3.42	2.90	2.91	4.28	3.58	3.32	4.69	4.03
ATC Class J	5.92	9.46	11.12	8.33	10.44	11.68	9.34	10.69	12.61	9.84	11.85	13.19	10.93	13.28	15.33
ATC Class L	0.60	1.29	1.58	0.89	1.10	1.32	1.01	1.05	1.44	0.98	1.57	1.58	1.09	2.19	1.53
ATC Class M	3.17	8.29	9.63	6.46	10.13	10.05	8.61	12.31	12.17	9.64	13.38	14.03	9.85	12.18	14.92
ATC Class N	14.57	36.11	46.90	24.76	38.15	43.08	31.31	40.43	50.24	33.33	37.97	54.66	37.10	49.16	54.84
ATC Class P	0.00	0.00	0.00	0.00	0.00	0.00	0.00	0.00	0.00	0.00	0.00	0.00	0.00	0.00	0.00
ATC Class R	7.02	14.12	16.31	11.51	16.09	17.10	15.43	20.84	20.65	16.60	24.29	23.60	19.12	28.30	28.26
ATC Class S	0.84	2.86	4.11	1.77	3.52	3.92	1.92	3.28	3.92	1.63	2.86	3.74	1.50	3.08	3.47
**Diagnostics - Visits**	**102.7**	**299.6**	**278.4**	**136.3**	**308.3**	**246.3**	**174.1**	**329.5**	**282.3**	**170.3**	**334.3**	**279.8**	**171.2**	**324.7**	**281.1**
Laboratory Diagnostics	29.8	98.49	86.47	35.46	97.85	75.49	43.80	100.4	85.54	44.11	105.5	83.72	46.87	99.52	83.76
Non-Laboratory Diagnostics	61.2	173.5	162.2	86.99	180.4	142.9	113.5	199.9	169.3	110.7	194.4	167.0	103.9	185.7	165.3
Check-up Visits	1.46	4.39	7.12	2.41	5.53	6.44	2.80	5.57	6.73	2.81	6.02	6.44	3.07	5.55	6.63
Specialist Visits	5.36	13.54	12.91	6.88	14.79	11.73	8.02	14.99	12.01	8.18	15.79	12.23	8.89	15.45	12.29

* Class A: alimentary tract and metabolism; class B: blood and blood forming organs; class C: cardiovascular system; class D: dermatologicals; class G: genito urinary system and sex hormones; class H: systemic hormonal preparations, excluding sex hormones and insulins; class J: antiinfective for systemic use; class L: antineoplastic and immunomodulating agents; class M: musculo-skeletal system; class N: nervous system; class P: antiparasitic products, insecticides and repellents; class R: respiratory system; class S: sensory organs.

### The interplay between excess weight and blood glucose abnormalities: impact on outpatient healthcare expenditure

Table 5 shows the average per capita costs by BMI classes and glucose metabolism status. Within each BMI class, the presence of IFG or DM2 identified subgroups of individuals with a substantially higher healthcare expenditure (figure S2). The presence of glucose metabolism alterations was associated with increased drug expenditures, particularly in ATC classes A, C, B, and N, in all BMI classes. The drugs expenditure ratio for individuals with IFG and those with DM2 as compared to those with normal blood glucose levels was particularly high for normal-weight individuals, with a more than sixfold higher expenditure associated with DM2 and a more than three times higher cost for individuals with IFG, compared to those with normal glucose levels. In the other BMI classes, drug expenditure was more than two times higher in individuals with DM2 and 50–90% higher in those with IFG than individuals with normal glucose levels. As for costs associated with visits or diagnostic tests, the highest average values were reported for people with IFG in all BMI classes.

### Estimate of potential cost savings

Finally, for 2018 we estimated the outpatient cost savings associated with the potential weight loss of the Italian population toward the normal-weight class. In particular, our simulation suggests saving about 73 million euros annually if all overweight individuals move to the normal-weight class. This amount increases to 213, 362, and 553 million euros, respectively, if they could move from the first, second, and third class of obesity to the normal-weight level. Overall, the yearly savings in outpatient expenditure sum up to 1.2 billion euros. Interestingly, the largest share of these savings is obtained among individuals aged 35–55, the age bracket where chronic diseases are not yet supposed to be present unless body weight problems occur.

## Discussion

### Major findings

In this study, we evaluated the disease burden and the effects of BMI on outpatient health care costs, in a large population of 991,917 adult men and women for whom accurate anthropometric, clinical and medical cost data collected by general practitioners were available.

The analysis of this extensive clinical database showed that excess body weight is widespread in Italy, affecting half of the adult population. Our findings are in line with recent WHO estimates, according to which one in two citizens in Europe is overweight or obese [20].

We confirmed the elevated clinical burden conferred by excess weight: both overweight and obesity were associated with an increasing prevalence of a large array of different chronic conditions, including cardiovascular diseases, chronic kidney disease, osteoarticular diseases, depression, and sleep apnea. This translated into a substantially higher outpatient healthcare expenditure. We found that costs associated with drugs and outpatient services (visits and diagnostic procedures) markedly increased with increasing BMI in all age categories; the impact is particularly strong among individuals below 65 years of age. Overweight and obesity were associated with a striking increase in the costs associated with drugs of the cardiovascular system, drugs of alimentary tract and metabolism, and drugs of the nervous system, with the largest cost ratios vs. normal weight being present for drugs of the cardiovascular system.

These results are extremely important from a public health perspective. The highest cost increase is among the young patients, who are those for whom avoiding excess weight would produce the highest benefits. These patients could avoid drug treatment if they were normal-weight, which may not happen when getting older. Having overweight or obesity for these patients means also longer period of treatment and higher chances in the future of adding comorbidities and health problems, and more frequent access to health care services. In other words, whatever happens below age 65 could be easily preventable, which is why the cost ratios are higher.

Glucose metabolism alterations are mainly linked to overweight and obesity [21]: in our study population, diabetes was registered in one in seven people with normal weight, one in four overweight individuals, one in three individuals with obesity class 1 and 2, and four in ten individuals with severe obesity. The presence of diabetes strongly influenced the likelihood of suffering from other chronic conditions [12]. In addition, a pre-diabetes status indicated by impaired fasting glucose was also associated with an elevated risk of significant comorbidities, suggesting a continuum in the risk related to glucose abnormalities. Within each BMI class, the presence of IFG or DM2 identified subgroups of individuals with a substantially higher outpatient healthcare expenditure.

### Implications for clinical practice

Our study provides an up-to-date estimate of the clinical and economic burden of excess weight in Italy. It confirms the urgent need to intervene to limit the growth of the obesity pandemic. The study shows that even moderate increases in body weight, in the range of overweight, are associated with an increased healthcare expenditure, thus suggesting the need to intervene promptly and effectively to counteract weight gain. The study also shows that the concomitance of excess weight and glucose metabolism alterations, even before overt diabetes, further increases outpatient expenditure. Thus, a proactive approach is needed to identify glucose metabolism alterations and address them with specific lifestyle and pharmacological interventions. Our data also emphasize the crucial role of primary prevention of obesity and hyperglycemia. These findings are significant for primary care, representing the forefront of the fight against obesity, diabetes, and related comorbidities.

### How could the results contribute to other countries beyond Italy?

We believe that these findings can be easily extended to other Western countries. This is because we have identified a “structural” connection between costs and a well-documented epidemiological, medical, and clinical issue. As long as BMI levels rise (Western countries are all facing an obesity epidemic), the expenses related to medications and outpatient services significantly increase across all age groups. Moreover, extensive clinical research has shown that the presence of glucose metabolism disorders greatly exacerbates medical issues and their associated costs. Lastly, when encountering these problems at a younger age, the consequences of being overweight or obese become even more pronounced. These relationships are in a sense “universal” and, therefore, easily extendable to other countries.

### Strengths and limitations

Among the major strengths of our study, we should mention the extensive sample, representative of the Italian adult population. The Italian National Health Service is a public and universalistic system that provides substantially free health services for all citizens. This setting favors the generalizability of the study and minimizes the selection problems related to the presence of private insurance plans. Furthermore, while many studies are based on health surveys with self-reported information, we used accurate anthropometric and clinical data collected by general practitioners, allowing a reliable estimate of the prevalence of obesity and related conditions, treatments, and outpatient resource utilization.

Among the limitations, we could not estimate inpatient costs since hospitalizations and their primary cause are not systematically registered in primary care medical records. Also, we could not estimate the costs relative to children and adolescents since this population is in charge of pediatricians not involved in our study. Finally, it is acknowledged that waist circumference represents a more accurate measure of visceral adiposity and obesity-related health risk than BMI [22]. However, in clinical records, waist circumference was not reported in a standardized and systematic way, precluding the possibility of using this measure in our study.

## Conclusions

In conclusion, one in two Italian adults have excess body weight, and one in ten have obesity. This has significant health consequences, which are reflected by increasing healthcare outpatient costs. Costs associated with drugs and outpatient services markedly increase with BMI in all age categories; the impact is particularly strong among younger individuals. Within each BMI class, the presence of glucose metabolism alterations substantially increases outpatient healthcare expenditure. Addressing the double burden of excess weight and hyperglycemia represents a significant challenge and a healthcare priority.

## Electronic supplementary material

Below is the link to the electronic supplementary material.

Supplementary Material 1

Supplementary Material 2

Supplementary Material 3

## Data Availability

The data that support the findings of this study are available from HealthSearch but restrictions apply to the availability of these data, which were used under license for the current study, and so are not publicly available. Data are however available from the authors upon reasonable request and with permission of HealthSearch.
