# Supplementary figures and images for: Outpatient healthcare costs associated with overweight and obesity in Italy
Source: BMC Health Serv Res. 2023 Jun 12;23:619. doi: 10.1186/s12913-023-09576-4 (PMC10258973; doi:10.1186/s12913-023-09576-4)

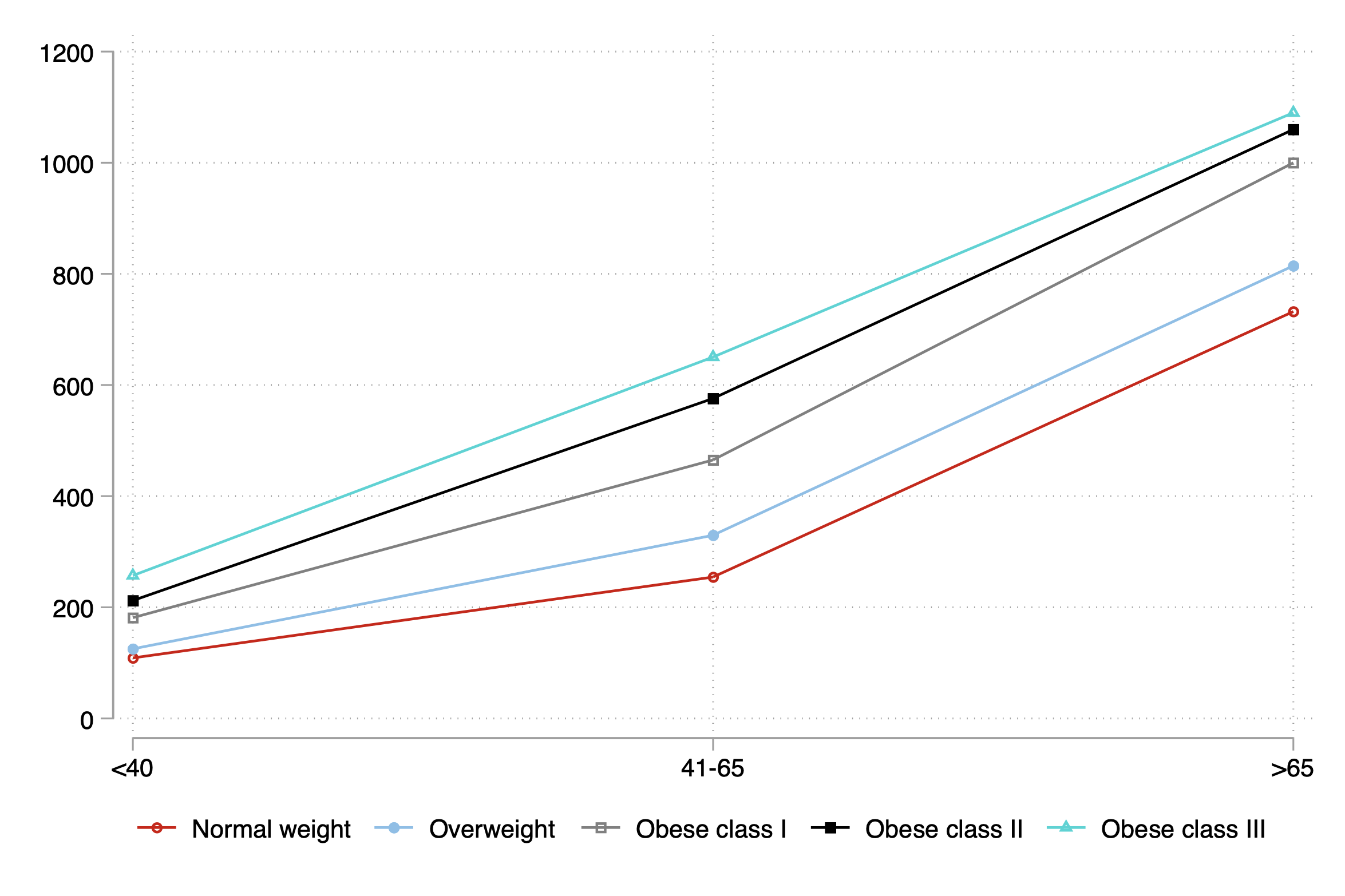

Supplement: Supplementary file 1 — Supplementary Material 1 [file 12913_2023_9576_MOESM1_ESM.png]

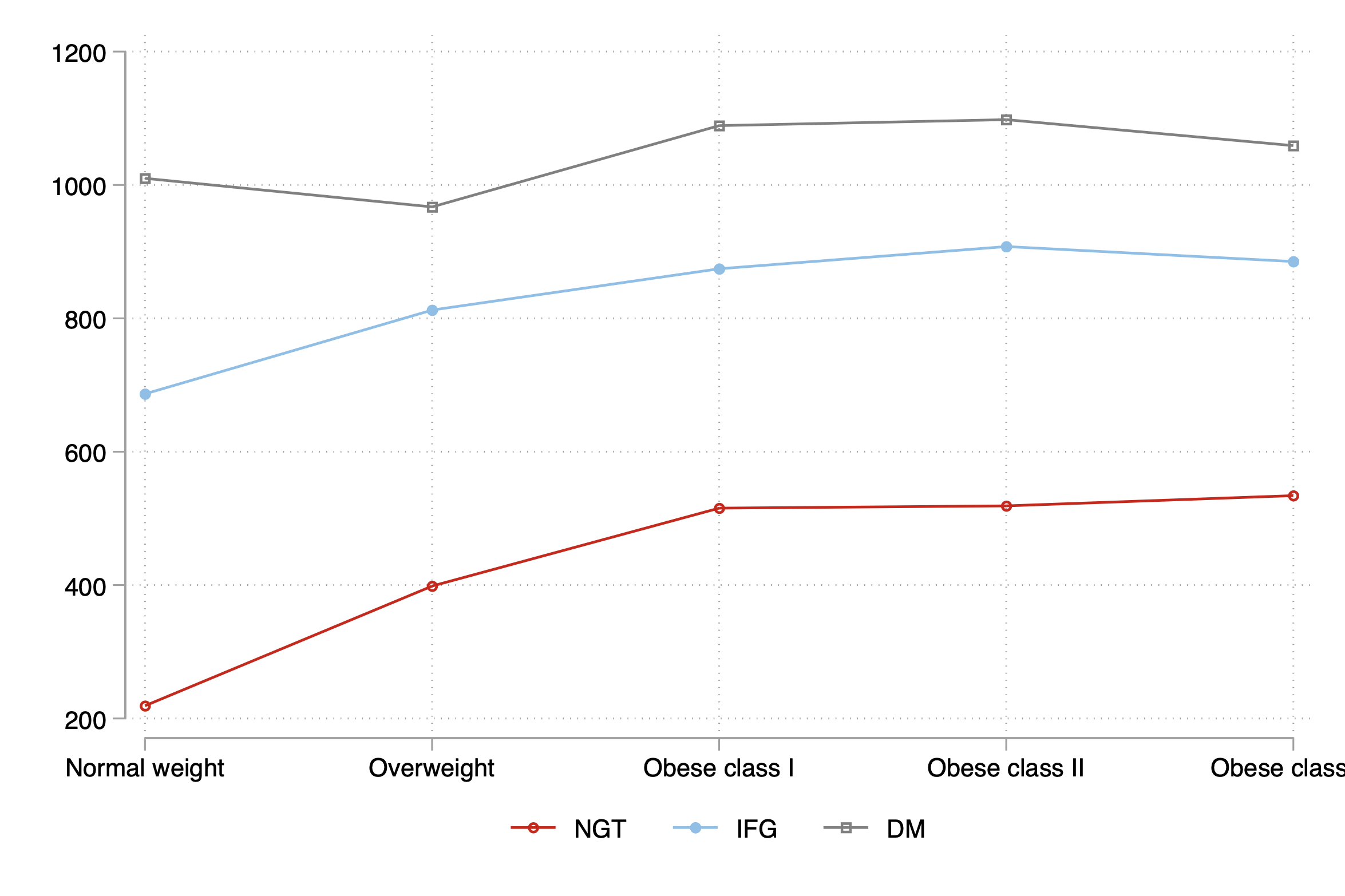

Supplement: Supplementary file 2 — Supplementary Material 2 [file 12913_2023_9576_MOESM2_ESM.png]
