# Supplementary material for: Outpatient healthcare costs associated with overweight and obesity in Italy
Source: BMC Health Serv Res. 2023 Jun 12;23:619. doi: 10.1186/s12913-023-09576-4 (PMC10258973; doi:10.1186/s12913-023-09576-4)
Supplement: Supplementary file 3 — Supplementary Material 3 [file 12913_2023_9576_MOESM3_ESM.docx]

Appendix 1. Missing imputation techniques adopted

Within the HS database, over the 2004-2018 period, there were 641,849 individuals with at least one value of BMI recorded. The lack of BMI records across patients and over time is rather common: if there are no medical and clinical reasons to measure height and weight, GPs tend not to routinely measure them at each visit. A second problem that interferes with the regular collection of BMI data is related to a selection process for which, whenever patients are not medically and clinically problematic in terms of BMI, GPs tend to collect this information less frequently. Therefore, one of the main threats to our analysis is the presence of missing values on BMI measurements, especially for the healthy population. Nonetheless, the sample still presents information on BMI levels (over time) for this problematic subgroup. Hence, by exploiting this information, and assuming that the sample selection generated by the missingness is driven by observable characteristics such as health status and age, we made an attempt to lessen the negative impact of the ascertainment bias on our analysis. Our objective was not to impute the BMI level to each subject, a too challenging task, but at least try to infer the BMI class to which each subject belongs. To this aim, we exploited a pooled ordered probit panel data model with sample selection in which the response is an ordered categorical variable indicating the BMI classes [18]. The latter have been constructed according to the following classification: 1) Normal weight [BMI 18.5-24.99], 2) Overweight [25.0-29*.*99], 3) Obese class 1 [30.0-34.99]; 4) Obese class 2 [35.0-39.99], 5) Obese class 3 [*≥* 40]^^[[1]](#footnote-1)^^.

Our statistical model for the data can be represented as follow:

$$y_{j,it}^{*} =\boldsymbol{x}_{j,it}\beta_{j}+\epsilon_{j,it} j=1,2 (1)$$

$$y_{1,it}= I(y_{1,it}^{*}>0) (2)$$

$$y_{2,it}=\sum_{h=1}^{H} hI(\alpha_{h}<y_{2,it}^{*}\leq\alpha_{h+1}) if y_{1,it}=1 (3)$$

where $y_{1,it}^{*}$ and $y_{2,it}^{*}$represent, for each subject *i* and year *t*, continuous latent variables for the selection process and the BMI outcome, respectively, the $\boldsymbol{x}_{1,it}$and $\boldsymbol{x}_{2,it}$are vectors of exogenous of individual characteristics, such as age, gender and region of residence, the fraction of individuals whose BMI was effectively recorded for year *t* at GP level and year fixed-effects. $\boldsymbol{x}_{2,it}$ also includes a vector of selected diseases indicators related to obesity (cerebrovascular diseases, coronary diseases, heart failure, peptic ulcer disease, diabetes mellitus) and solid tumor (esophagus, stomach, intestine, colon, rectum, liver, gallbladder, pancreas). Finally, the $\epsilon_{1,it}$ $\epsilon_{2,it}$ are idiosyncratic errors, $\beta_{j}, j=1,2$, are the vectors of parameters to be estimated.

As in a standard ordered probit model, the (latent) BMI $y_{2,it}^{*}$ is assumed to be linked to the observed categorical variable $y_{2,it}$ (BMI classes) through the observation rule (3), where $\delta=(\delta_{0},\ldots,\delta_{H})$, with $\delta_{h}\leq\delta_{h+1}$ is a vector of unknown thresholds that partition $y_{2,it}^{*}$into $H+1$ mutually exclusive BMI classes. Due to the missing data (selection mechanism), the $y_{2,it}$ variable is observed only for the subsample of observation for which $y_{1,it}=1$ i.e. the selected sample.

Identification of the unknown parameter vectors $\beta_{j}, j=1,2$ and $\delta$ requires an exclusion restriction in the selection equation (2), that $\boldsymbol{x}_{1,it}$must contain at least one variable that is not contained in $\boldsymbol{x}_{2,it}$.^^[[2]](#footnote-2)^^

We argue that the selection mechanism is driven by health status, thus we consider as exclusion restriction two binary indicators for being healthy and under 40 years.

The joint distribution of $\epsilon_{1,it}$ $\epsilon_{2,it}$ is assumed to be bivariate normal with zero mean, ${Var(\epsilon}_{1i})= \mathrm{Var}(\epsilon_{2i})=1$, and $Cov\left( \epsilon_{1,it} \epsilon_{2,it} \right)=\rho.$ We estimated model (1)-(3) by pooling the unbalanced panel of 1,498,598 individuals aged [18-95] over the 15 year period.^^[[3]](#footnote-3)^^ We then used the estimated parameters to estimate the probability that $y_{2,it}=h$ given that $y_{2,it}$is not selected, that is:

$Pr(y_{2,it}\hat{={h|y}_{1,it}}= 0)=\frac{\Pr{(y}_{2,it}\hat{={h,y}_{1,it}}= 0)}{Pr(\hat{y_{1,it}}= 0)}$ (4)

where

$$\Pr\left( y_{2,it}\hat{={h,y}_{1,it}}= 0 \right)=\Phi_{2}\left( -\boldsymbol{x}_{2,it}\hat{\beta_{2},}\delta_{h}-\boldsymbol{x}_{1,it}\hat{\beta_{1},}\rho\right)-\Phi_{2}\left( -\boldsymbol{x}_{2,it}\hat{\beta_{2},}\delta_{h-1}-\boldsymbol{x}_{1,it}\hat{\beta_{1},}\rho\right) (5)$$

with $\Phi_{2}$ the cumulative bivariate normal distribution function (with mean [0,0]*'*) and the probability that the outcome$y_{2,it}=h$ is not selected is $\Pr\left( \hat{y_{1,it}}=0 \right)=1- \Phi\hat{(\boldsymbol{x}_{1,it}\hat{\beta_{1},}})$. We then assigned the missing BMI class to subject *i* in year *t* accordingly, i.e. $\hat{y}_{2,it}=h$ if $\Pr{(y}_{2,it}\hat{={h|y}_{1,it}}= 0)<$ $\Pr\left( y_{2,it}\hat{=j|}y_{1,it}= 0 \right)\forall j\neq h$.

Appendix 2. ICD-9 CM codes used to identify comorbidities.

| Comorbidity | ICD-9 CM codes |
| --- | --- |
| Diabetes mellitus | 250 |
| Coronary heart disease (myocardial infarction, coronary reperfusion/revascularization) | 410, 412, v45.81, v45.82 |
| Cerebrovascular disease (stroke, transient ischemic attack, carotid artery revascularization) | 431, 433.00, 433.01, 433.11, 433.21, 433.31, 433.81, 433.91, 434.01, 434.11, 434.91, 435, 436, 438, v45.89 |
| Heart failure | 428, 402.0, 402.01, 402.11, 402.91, 404.00, 404.03, 404.11, 404.13, 404.91, 404.93 |
| Osteoarticular diseases (hip and knee osteoarthrosis) | 715.35, 715.95, 715.36, 715.96 |
| Depression | 311, 296.2, 296.3 |
| Chronic kidney disease | 585 |
| Cancer (esophagus, stomach, intestine, colon, rectum, liver, gallbladder, pancreas) | 150, 151, 152, 153, 154, 155, 156, 157 |
| Sleep apnea | 780.51, 780.53, 780.57 |
| PCOS | 256.4 |

1. For the sake of brevity but without any loss of information, we do not report the underweight class (BMI below 18.49) as not relevant for our analysis. [↑](#footnote-ref-1)
2. We also note that weak identification of the parameters is still possible even without a strong exclusion restriction due to the non-linearity of the model. See Wilde [19] for the intuition behind this claim. [↑](#footnote-ref-2)
3. The full set of estimation results is available upon request. [↑](#footnote-ref-3)
